# Sensemaking in the formation of basic life support teams - a proof-of-concept, qualitative study of simulated in-hospital cardiac arrests

**DOI:** 10.1186/s13049-018-0479-0

**Published:** 2018-01-29

**Authors:** Peter Hallas, Johnny Lauridsen, Mikkel Brabrand

**Affiliations:** 1 Institute of Regional Health Research, University of Southern Denmark, Finsensgade 35, 6700 Esbjerg, DK Denmark; 2 Department of Emergency Medicine, Zealand University Hospital, Lykkebækvej 1, 4600 Køge, DK Denmark; 3 Department of Anaesthesiology and Intensive Care, Svendborg Hospital, Baagøes Allé 15, 5700 Svendborg, DK Denmark; 4 Department of Emergency Medicine, Hospital of South West Jutland, Finsensgade 35, 6700 Esbjerg, DK Denmark

**Keywords:** Cardiac crash team, Social interaction

## Abstract

**Background:**

The formation of critical care teams is a complex process where team members need to get a shared understanding of a serious situation. No previous studies have focused on how this shared understanding is achieved during the formation of cardiac arrest teams. “Sensemaking” is a concept well known in organizational studies. It refers to the collaborative effort among members in a dialogue to create meaning in an ambiguous situation, often by using subtle variations in the sentences in the dialogue. Sentences with high degrees of “sensemaking” activity can be thematized as “co-orientation”, “re-presentation” and/or “subordination” (among others). We sought to establish if elements of “sensemaking” occur in the formation of in-hospital cardiac arrest teams.

**Methods:**

Videos of ten simulations of unannounced in-hospital cardiac arrests treated by basic life support (BLS) providers. We transcribed all verbal communication from the moment the first responder stepped into the room until the moment external chest compression were initiated (verbatim transcription). Transcriptions were then analyzed with a focus on identifying three elements of sensemaking: Co-orientation, Re-presentation and Sub-ordination.

**Results:**

Sensemaking elements could be identified in seven of ten scenarios as part of team formation. Co-orientation was the element that was used most consistently, occurring in all of the eight scenarios that included sensemaking efforts.

**Conclusions:**

Sensemaking is an element in the communication in some cardiac arrest teams. It is possible that the active moderation of sensemaking should be considered a non-technical skill in cardiac arrest teams.

## Background

The quality of cardiopulmonary resuscitation is closely linked to team dynamics and team leadership of the cardiac arrest team [1] with non-technical leadership skills such as structured communication and a focus on team coordination having a key role [2, 3]. The team formation process is therefore crucial for performance of the cardiac arrest team. However, the formation of critical care teams is a complex process that can be challenging because team members might not have a shared understanding of the situation and because communication in situations under time pressure can be challenging [4].

In organizational studies, the concept of sensemaking refers to the process where more individuals gain a common sense of understanding of situations [5]. Sensemaking” *involves turning circumstances into a situation that is comprehended explicitly in words and (…) serves as a springboard into action*” [5]. Thus, sensemaking involves ongoing communication with-in and between individuals working together. Studies have documented the role of sense-making in a number of aspects in health care, from the strategic levels [6] to disaster management [7] and errors in medicine [8]. Active moderation of the sensemaking process has been suggested as a leadership tool in team leadership [9]. However, no previous studies have examined if sense-making processes occur in the formation of cardiac arrest teams.

In this proof-of-concept study we sought to establish if sense-making occur as part of the formation of the cardiac arrest teams in in-hospital cardiac arrests treated by basic life support (BLS) providers.

## Methods

This analysis used data from a simulation study of unannounced in-hospital cardiac arrests. The project was carried out in 2012 and 2013 at eight wards in four hospitals in the Region of Southern Denmark as part of a local health technology assessment project. The simulations used a Resusci Anne Simulator (Laerdal Medical®) in a cardiac arrest scenario.

Each scenario was prepared in a vacant room at a participating ward without actively notifying the ward staff (The ward staff had been notified with posters at the wards, that the project was ongoing but did not know on which day the scenario would take place at their ward). When the equipment was ready, the scenario moderator used the room’s emergency button to summon staff, in the same manner as a patient would if calling for help. When the staff arrived, an identical scenario was played: the patient was responsive but moaning, takypneic and uttering a few words (“*I don’t feel so well*”). After 30 s, the patient became apneic and unresponsive as a sign of cardiac arrest. After 8 min, the patient had return of spontaneous circulation and breathing regardless of treatment. All scenarios (*n* = 49) were video recorded.

Participants had consented to participation and the project was approved by the Danish Data Protection Agency. Other results from the health technology assessment project are reported elsewhere.

Consecutive videos were screened until 10 videos could be included as a proof-of-concept. Videos could be included if more than one BLS provider was present and if the technical aspects of the video allowed analysis of the dialogue. A total of 14 videos were screened.

For each video, the conversation between the BLS providers was transcribed starting from the moment that the first responder stepped into the room until the moment external chest compressions were initiated. This interval was chosen to illustrate an initial, time-critical interval where there needs to be “*a springboard into action”* by the team (action in the form chest compressions). The transcription was done by one author (PH) and focused not only on what was said, but also on small interruptions and breaks in conversations (verbatim transcript). This approach is known from the field of conversation analysis [10] albeit used here in a modified version (e.g. breaks were not measured in milliseconds).

The transcriptions were analyzed with focus on identifying three elements in the construction of sense-making in conversations [10, 11]:

**Co-orientation** - A contribution that confirms the object of conversation.

**Re-presentation** - literally the presentation again of past interactions.

**Sub-ordination** – alignment of a contribution to the conversation within the premises of a previous statement.

The analysis was performed by looking at each sentence in the conversation and categorizing its relationship to the sentence immediately before - using the three categories above. If a sentence could not be included in of the three a priori categories, it was not categorized.

One author (PH) did the primary analysis of all the videos. Transcripts and the preliminary results of the analysis were then shared with another author (MB) and discrepancies in interpretations were discussed. Only categorizations where there a shared understanding could be achieved are included. The study follows the Standards for Reporting Qualitative Research [12].

It should be noted that the staff communication was in Danish: Thus, for the purpose of this paper, the conversations are translated into a modified verbatim version in English. Interruptions by the next person speaking is annotated with // at the beginning of the interruption. The responders are numbered in the order that they entered the room in the scenario.

## Results

Sensemaking elements could be identified in eight of ten scenarios as part of team formation (Table 1). Co-orientation was the element that was used most consistently, occurring in all of the eight scenarios that included sensemaking efforts (Table 1). Median number of team members were 4 (range 2–5).

**Table 1:** Examples of sensemaking elements in the team formation

Element	Scenarios with element (total of 10 scenarios)	Examples
Co-orientering	8	*Responder Three: “He has a pulse”* *Responder One “He is breathing too…”* *Unidentified Responder:“It is just a drill”* *Responder One: “Yeah //”*
Re-presentation*	6	*Responder One:“Yes. There is cardiac arrest”* *Responder Two “There is arrest?!”* *Responder One: He ain’t breathing* *Responder three:*” *No he ain’t”*
Sub-ordination	7	*Responder One:“There is arrest now, yes”* *Responder Two “w’ll active the alarm, then”* *Unidentified Responder: It isjust a drill* *Responder One: “Yeah //”* *Responder three: “yes”//* *Responder One (laughing):* *I’m getting all scared*
No elements of sense making	2	*Responder one: I want you to get the medsand active the alarm in the hallway and get another nurse in here. (pause)* *Are you there?* *Responder two: Coming now*

Many of the verbal exchanges between the BLS providers could be seen as having dual purposes, i.e. could be seen as both e.g. “co-orientation AND re-representation” or “re-presentation AND sub-ordination”. These cases are counted a belonging to both categories in the results.

In the two scenarios were no sense making dynamics could be detected as there were little or no communication between responders in the observed interval.

Two scenarios can further illustrate the dynamics of team formations seen in the analysis. This can be illustrated in the transcript below, which is from a scenario with four responders.

**Table:** 

Responder-ID	Transcript	Interpretation
Responder one	*Hallo*	
Responder two	*Gosh!*	
Responder two	*He is brea…is he breath//ing?*	
Silence 5 s.		
Responder one	*He is breathi he is breathing*	Co-orientation
Responder two	*But really compromised*	Subordination
Responder one	*Well now, but he is breathi//ing*	Re-presentation
Responder two	*Real fast*	Subordination
Responder two	*Can anyone get a doctor inhere*	
Responder three	*Get a doctor?... yea//h*	
Responder four	*Yes*	
Silence 2 s		
Responder four	*Well but, he is breathing* *Should//n’t we ventilate get//*	Re-presentation
Responder three	*Is there something I should*	
Responder two	*And also the crash cart*	
*Silence 4 s.*		
Responder two	*Well, now he is not breathing anymore* *we will try*	
Responder one	*Take out*	
Chest compressions begun		

In this example, sense-making elements occur around a dialogue about the breathing pattern and a common understanding brings the actions forward, eventually towards recognition of the cardiac arrest when it occurs. This team dynamic can be compared to another scenario where the lack of development in sensemaking between the responders seems to stall the resuscitation:

**Table:** 

Responder-ID	Transcript	Interpretation
Responder Two	*I’ll get the …crash cart*	
Responder One	*No ‘cause he is breathing*	
Responder Two	*Yes* *You can see…he is breathing*.. *Yeah*	Re-presentation
Responder One	*Seems somewhat pale*	
Silence 6 s		
Responder Three	*What has happened?*	
Responder (ID?)	*The cart?*	
Responder Two	*We need t//*	
Responder Three	*Ar//rest*	
Responder One	*Ar//rest*	Co-orientering
Responder Two	*There is arrest*	
Responder (ID?)	*Are you getting help?*	
Responder One	*He is not breath*	Re-resentation/ subordination
Responder Three	*No he is not*	Co-orientation
Responder One	*There is no pulse*	Re-presentation
Chest compressions begun		

In this scenario, Responder One blocks the implicit suggestion of fetching the crash cart with the words “*No ‘cause he is breathing”*. Responder Two then offers a re-representation of the rationale for this, but Responder One does not follow up and stays silent; (This leaves Responder Two with the only option of herself confirming what she just said: “*yeah*”).

Then the situation reverses: responder one offers a “*seems somewhat pale*” as a starting point for a dialogue - but now Responder Two does not follow the lead and stays silent. They do not initiate chest compressions even though they all acknowledge that there is cardiac arrest. This stalemate does not resolve until a third participant (Responder (Unidentified)) comes by and interrupts. In this scenario, the actions around the patient seem to stall because a sensemaking action is not “played out”.

The two scenarios are somewhat typical of the scenarios included in this subset of videos: Despite the tense and serious situation (a cardiac arrest), the participants engage in a sort of micro-discussion to identify the action required and to identify who is in control. This illustrates that when sensemaking elements are identified, a focus on the micro-informational exchange between team members gives further insight into the dynamics of team formation.

## Discussion

This proof-of-concept study establishes sensemaking as an element in the communication in some cardiac arrest teams and shows that tacit communication strategies within cardiac arrest teams can be analyzed using the themes of co-orientation, re-presentation and subordination. Thus, it is possible that the moderation of sensemaking should be considered a non-technical skill in cardiac arrest teams.

The use of sensemaking in critical situations might be a rational way of dealing with situational ambiguity and promote team resilience [13, 14]. From a theoretical point of view, sensemaking has been seen as part of a process dubbed “collective minding”, i.e. a situation where it is the shared knowledge that decides the turn of actions [11]. Thus, the present study deepens the understanding of team dynamics in cardiac arrest teams and suggests that the process of team formation goes beyond establishing a leadership role and giving commands to a complying team, at least for BLS providers. However, as can be seen from Table 1, not every scenario went through all elements in the sense making process as defined here. Although beyond the scope of a pilot-study, this variation in team communication patterns could be a clue to valuable insights into team dynamics [15].

A bias in this study is that although the study focused on unannounced simulations, the ward staff could sometimes tell in advance that a simulation event would take place, e.g. if they spotted one of the scenario moderators at the ward before the simulation. Thus, it is possible that in some cases, part of the team formation process will have taken place prior to the first person entering the room (and therefore not recorded on video). Another possible bias is the subtle nature of the elements studied. It takes some training to transcribe and analyze the conversations and there will invariably be an element of inter-observer variation. We have sought to minimize that by having more people analyze the transcripts and by only including elements were the two raters independently could agree on a categorization. Finally, only a limited number of simulations were included (without data saturation being reached); including more of the simulations in further studies might reveal another pattern.

Cooren [12, 16] showed that subtle cues to an ongoing sense making process could be detected on the” micro-level” in an everyday situation. His classical study showed that sense-making embedded in everyday conversation was part of an ongoing negotiation of power and goals between participants in conversations. Splitting the concept of sensemaking in cardiac arrest team communication into three distinctive categories makes the concept accessible for analysis and perhaps even training. This also raises some questions: Does sense-making influence team for performance? Can sensemaking be actively moderated as a non-technical skill? Further studies should look into this.

## Conclusion

Sensemaking is an element in the communication in some cardiac arrest teams. It is possible that the moderation of sensemaking should be considered a non-technical skill in cardiac arrest teams.
